# The epidemiology of acute gastrointestinal illness in Ethiopia, Mozambique, Nigeria, and Tanzania: a population survey

**DOI:** 10.1017/S095026882500038X

**Published:** 2025-04-21

**Authors:** Binyam N. Desta, Sara M. Pires, Tine Hald, Tesfaye Gobena, Custodia Macuamule, Belisario Moiane, Olanrewaju E. Fayemi, Christianah I. Ayolabi, Gabriel Akanni, Blandina T. Mmbaga, Kate M. Thomas, Happiness Kumburu, Warren Dodd, Shannon E. Majowicz

**Affiliations:** 1 School of Public Health Sciences, University of Waterloo, Waterloo, ON, Canada; 2 Risk-Benefit Research Group, Technical University of Denmark, Lyngby, Denmark; 3 Research Group for Genomic Epidemiology, Technical University of Denmark, Lyngby, Denmark; 4 College of Health and Medical Science, Haramaya University, Haramaya, Ethiopia; 5 Faculty of Veterinary, Eduardo Mondlane University, Maputo, Mozambique; 6 Centre for Research, Innovation, and Collaboration/Department of Biological Sciences, Mountain Top University, Prayer City, Nigeria; 7 Department of Microbiology, University of Lagos, Lagos, Nigeria; 8 Kilimanjaro Clinical Research Institute, Kilimanjaro Christian Medical Centre, Moshi, Tanzania; 9 Kilimanjaro Christian Medical University College, Moshi, Tanzania; 10 Centre for International Health, Dunedin School of Medicine, University of Otago, Dunedin, New Zealand

**Keywords:** diarrhoea, epidemiology, estimating disease prevalence, gastrointestinal infections, infectious disease epidemiology

## Abstract

Gastrointestinal infections significantly impact African low- and middle-income countries, although, accurate data on acute gastrointestinal illness (AGI) for all ages are lacking. This study aimed to describe the epidemiology of AGI in Ethiopia, Mozambique, Nigeria, and Tanzania. A population survey was conducted in one urban and one rural site per country, from 01 October 2020 to 30 September 2021, using web-based and face-to-face tools (n = 4417). The survey tool was adapted from high-income countries, ensuring comparability through an internationally recommended AGI case definition. Ethiopia had the highest AGI incidence (0.87 episodes per person-year), followed by Mozambique (0.58), Tanzania (0.41), and Nigeria (0.34). Age-standardized incidence was highest in Mozambique (1.46) and Ethiopia (1.25), compared to Tanzania (0.58) and Nigeria (0.33). The 4-week prevalence was 6.4% in Ethiopia and 4.3% in Mozambique, compared to 3.1% in Tanzania and 2.6% in Nigeria. AGI lasted an average of 5.3 days in Ethiopia and 3.0 to 3.4 days elsewhere. Children under five had 4.4 times higher AGI odds (95% CI: 2.8, 6.7) than those aged 15-59. The study provides empirical data on the incidence and demographic determinants of AGI in these four countries.

## Introduction

Gastrointestinal infections transmitted by food are a global concern [1], and low- and middle-income countries (LMICs) bear the highest burden [2]. These infections are severe in Africa, illustrated by the estimated 1 in 10 child and 1 in 13 all-ages deaths caused by diarrhoea [2, 3]. Aggregate-level estimates indicate that the diarrhoeal disease burden in African LMICs is higher than that of malaria, preterm birth complication, and tuberculosis and is comparable to HIV/AIDS [3, 4].

To date, data on acute gastrointestinal infections (AGIs) in African LMICs have overwhelmingly come from children under 5 years, including from national Demographic and Health Surveys (DHSs) that report the 2-week prevalence of diarrhoea for this age group (e.g., [5–13]). While many countries globally have conducted surveys to study the epidemiology of AGIs in the general population for all ages (e.g., [14–21]), in Africa such studies are limited to an Indigenous community in Uganda [22] and an urban setting in South Africa [23]. Thus, except for the World Health Organization (WHO)‘s aggregate-level estimates and crude reports of annual diarrhoeal cases [3], population incidence estimates of AGI are lacking. Moreover, having incomparable national incidence and prevalence estimates (i.e., 2-week versus 4-week recall; varying case definitions) limits a country’s ability to compare its achievements in the control of AGI with others internationally, makes it difficult to contribute to international research, and can affect the country’s representation when international-level policies are made [24]. Thus, this study aimed to describe the epidemiology of AGI at the population level in Ethiopia, Mozambique, Nigeria, and Tanzania, specifically to estimate the incidence, prevalence, and duration of AGI and identify demographic determinants.

## Methods

### Study design

A cross-sectional population survey, collecting self-reported data on the occurrence of AGI, was conducted from 1 October 2020 to 30 September 2021 in Ethiopia, Mozambique, Nigeria, and Tanzania. The target population was the general population in each of the four countries, and the study was conducted in one urban and one rural site per country. Full details about study sites, sample sizes, ages of assent, remuneration, survey languages and formats, timing of data collection, ethics boards (with certificate numbers), and DOI links to the survey tools are given in Supplementary Material, Table S1.

Briefly, the minimum target sample size was 372 per site (calculated assuming a > 10000 population size, 10% estimated AGI prevalence, 3.1% allowable error, 95% certainty). From this minimum, target sample sizes then varied by country, due to the time it took to go to the study sites and from house to house and the cost of enumerators’ time. Respondents provided informed assent/consent. Those 18 years and older answered the survey themselves. Parents provided consent and answered the survey for young children. Youth provided assent in addition to parental consent and could choose to answer the survey themselves or have a parent answer. Specific ages varied by country. Nine research ethics boards in five countries approved this study.

### The survey tool

The survey was designed using standard questions from other AGI population surveys conducted in Canada, Denmark, Germany, Ireland, Italy, and New Zealand [14–19] and predominantly used closed-ended questions with predefined responses. The survey was developed in English [20] and then translated by fluent expert co-authors, into five other languages commonly spoken in each country [25]. Research team members reviewed survey drafts to ensure suitability for the study communities, and translations were cross-checked for content accuracy by independent reviewers from each study country.

The survey collected information on: diarrhoea (defined as any loose stool or stool with abnormal liquidity) or vomiting in the 4 weeks prior to the survey date; the date the illnesses started; the number of days the illnesses lasted; age; gender (measured as male/female: a culturally-appropriate composite measure of sex and gender widely understood by the study populations); wealth index (measured by questions on housing conditions, water supply and sanitation conditions, and property/material ownership); employment status of the main earner in the household; number of people sleeping per room; urban/rural status; country; and the date the survey was completed. Survey completion date was used to create the variable season, which followed each country’s dry and wet seasons (Table 1).

**Table 1.** Demographic characteristics of survey respondents and 4-week prevalence of acute gastrointestinal illness (AGI) in Ethiopia, Mozambique, Nigeria, and Tanzania (October 2020–September 2021; n = 4417)

Variable	Overall		Ethiopia		Mozambique		Nigeria		Tanzania	
	Number (%)	Prevalence (95% CI^a^)	Number (%)	Prevalence (95% CI^a^)	Number (%)	Prevalence (95% CI ^a^)	Number (%)	Prevalence (95% CI ^a^)	Number (%)	Prevalence (95% CI ^a^)
Gender										
Male	1718 (39.0)	3.9 (3.7, 4.2)	172 (21.7)	8.7 (4.8, 12.6)	343 (36.3)	4.1 (2.2, 6.0)	602 (42.7)	3.0 (1.8, 4.1)	601 (47.7)	3.5 (2.2, 4.8)
Female	2688 (61.0)	3.7 (3.5, 3.9)	619 (78.3)	5.8 (4.1, 7.5)	601 (63.7)	4.5 (3.0, 6.0)	808 (57.3)	2.3 (1.5, 3.2)	660 (52.3)	2.7 (1.7, 3.8)
Total	4406 (100)	3.8 (3.6, 4.0)	791 (100)	6.4 (4.9, 8.0)	944 (100)	4.3 (3.2, 5.5)	1410 (100)	2.6 (1.9, 3.3)	1261 (100)	3.1 (2.3, 3.9)
Age (years)										
0–4	508 (11.7)	9.4 (8.7, 10.2)	122 (15.6)	16.4 (10.4, 22.4)	24 (2.6)	12.5 (0.6, 24.4)	308 (22.2)	5.8 (3.6, 8.0)	54 (4.4)	13.0 (5.2, 20.7)
5–14	284 (6.6)	4.2 (3.5, 4.9)	13 (1.7)	15.4 (0.0, 33.4)	12 (1.3)	25 (3.0, 47.0)	189 (13.6)	2.6 (0.7, 4.6)	70 (5.7)	2.9 (0.0, 6.2)
15–59	3123 (72.2)	2.9 (2.7, 3.1)	546 (69.8)	4.4 (2.8, 6.0)	791 (85.6)	3.7 (2.5, 4.8)	848 (61.3)	1.4 (0.7, 2.1)	938 (75.9)	2.8 (1.9, 3.7)
60+	411 (9.5)	2.9 (2.4, 3.4)	101 (12.9)	4.0 (0.5, 7.4)	97 (10.5)	5.1 (1.2, 9.1)	39 (2.8)	2.6 (0.0, 6.7)	174 (14.1)	1.1 (0.0, 2.5)
Total	4326 (100)	3.8 (3.6, 3.9)	782 (100)	6.4 (4.8, 8.0)	924 (100)	4.3 (3.1, 5.5)	1384 (100)	2.6 (2.0, 3.3)	1236 (100)	3.0 (2.2, 3.8)
Mean	31.1		33.1		37.1		23.3		34.5	
Median	30.0		32.0		36.0		26.0		29.0	
Wealth index quintile										
Lowest	843 (20.4)	4.4 (4.0, 4.8)	153 (19.9)	4.6 (1.5, 7.6)	180 (19.9)	5.6 (2.5, 8.6)	272 (21.7)	2.9 (1.3, 4.6)	238 (19.9)	5.0 (2.6, 7.4)
Second & Middle^b^	1629 (39.5)	3.2 (2.9, 3.4)	311 (40.4)	6.1 (3.7, 8.5)	354 (39.2)	5.1 (3.0, 7.1)	484 (38.6)	1.0 (0.3, 1.8)	480 (40.1)	2.1 (1.0, 3.2)
Fourth	824 (20.0)	5.2 (4.8, 5.7)	152 (19.7)	9.9 (5.5, 14.2)	187 (20.7)	3.2 (0.9, 5.5)	244 (19.5)	4.5 (2.3, 6.7)	241 (20.1)	4.6 (2.3, 6.8)
Highest	830 (20.1)	3.8 (3.5, 4.2)	154 (20.0)	6.5 (2.9, 10.1)	183 (20.2)	3.3 (1.0, 5.6)	254 (20.2)	4.3 (2.2, 6.4)	239 (19.9)	2.1 (0.5, 3.6)
Total	4126 (100)	4.0 (3.8, 4.1)	770 (100)	6.6 (5.0, 8.2)	904 (100)	4.4 (3.2, 5.6)	1254 (100)	2.8 (2.0, 3.5)	1198 (100)	3.2 (2.3, 4.0)
Residence										
Urban	2221 (50.3)	3.0 (2.8, 3.2)	408 (51.6)	6.1 (4.0, 8.3)	426 (45.1)	3.5 (1.9, 5.1)	658 (46.4)	1.4 (0.6, 2.1)	729 (57.7)	2.3 (1.4, 3.3)
Rural	2196 (49.7)	4.6 (4.4, 4.9)	383 (48.4)	6.8 (4.5, 9.1)	519 (54.9)	5.0 (3.3, 6.7)	759 (53.6)	3.7 (2.6, 4.8)	535 (42.3)	4.1 (2.7, 5.6)
Total	4417 (100)	3.8 (3.6, 4.0)	791 (100)	6.4 (4.9, 8.0)	945 (100)	4.3 (3.2, 5.5)	1417 (100)	2.6 (1.9, 3.3)	1264 (100)	3.1 (2.3, 3.9)
Employment status of the main earner in the household										
Working	3220 (73.9)	4.1 (3.9, 4.3)	554 (70.2)	7.2 (5.2, 9.2)	706 (75.5)	5.1 (3.6, 6.6)	1086 (77.3)	2.9 (2.1, 3.8)	874 (71.2)	2.9 (1.9, 3.8)
Retired	265 (6.1)	3.4 (2.8, 4.0)	35 (4.4)	11.4 (1.8, 21.1)	60 (6.4)	3.3 (0.0, 7.4)	47 (3.3)	2.1 (0.0, 5.6)	123 (10.0)	1.6 (0.0, 3.5)
Student	385 (8.8)	3.4 (2.9, 3.9)	25 (3.2)	0.0 (0.0, 0.0)	30 (3.2)	6.7 (0.0, 14.7)	171 (12.2)	1.2 (0.0, 2.5)	159 (13.0)	5.7 (2.6, 8.7)
Housewife	427 (9.8)	2.3 (1.9, 2.8)	173 (21.9)	4.0 (1.3, 6.7)	116 (12.4)	0.9 (0.0, 2.4)	93 (6.6)	1.1 (0.0, 2.8)	45 (3.7)	2.2 (0.0, 5.9)
Disabled	42 (1.0)	4.5 (2.8, 6.3)	1 (0.1)	0.0 (0.0, 0.0)	17 (1.8)	0.0 (0.0, 0.0)	6 (0.4)	0.0 (0.0, 0.0)	20 (1.6)	10.0 (0.0, 21.3)
Others	14 (0.3)	7.1 (3.2, 11.0)	1 (0.1)	0.0 (0.0, 0.0)	6 (0.6)	0.0 (0.0, 0.0)	1 (0.1)	100.0 (-, -)	6 (0.5)	0.0 (0.0, 0.0)
Total	4355 (100)	3.9 (3.7, 4.0)	789 (100)	6.5 (4.9, 8.0)	935 (100)	4.4 (3.2, 5.7)	1404 (100)	2.6 (1.9, 3.3)	1227 (100)	3.2 (2.3, 4.0)
No. people sleeping per room										
≤ 3	3207 (75.6)	3.2 (3.1, 3.4)	257 (32.8)	5.1 (2.6, 7.5)	830 (90.5)	4.3 (3.1, 5.6)	1061 (76.8)	2.4 (1.6, 3.1)	1059 (91.4)	2.8 (2.0, 3.7)
> 3	1034 (24.4)	5.9 (5.5, 6.3)	527 (67.2)	7.2 (5.2, 9.2)	87 (9.5)	5.7 (1.3, 10.1)	320 (23.2)	3.7 (2.0, 5.5)	100 (8.6)	6.0 (2.0, 10.0)
Total	4241 (100)	3.9 (3.7, 4.0)	784 (100)	6.5 (4.9, 8.1)	917 (100)	4.5 (3.3, 5.7)	1381 (100)	2.7 (2.0, 3.4)	1159 (100)	3.1 (2.2, 4.0)
Mean	2.9		4.7		2.3		2.7		2.3	
Median	2.0		4.0		2.0		2.0		2.0	
(Range)	(1–15)		(1–15)		(1–14)		(1–9)		(1–7)	
Method of data collection										
Web-survey	1205 (27.3)	3.3 (3.0, 3.6)	45 (5.7)	4.4 (0.0, 1.0)	110 (11.6)	6.4 (2.3, 10.5)	607 (42.8)	1.5 (0.7, 2.3)	443 (35.0)	5.0 (3.2, 6.7)
Face-to-face	3212 (72.7)	4.0 (3.8, 4.2)	746 (94.3)	6.6 (4.9, 8.2)	835 (88.4)	4.1 (2.9, 5.3)	810 (57.2)	3.5 (2.4, 4.5)	821 (65.0)	2.1 (1.2, 2.9)
Total	4417 (100)	3.8 (3.6, 4.0)	791 (100)	6.4 (4.9, 8.0)	945 (100)	4.3 (3.2, 5.5)	1417 (100)	2.6 (1.9, 3.3)	1264 (100)	3.1 (2.3, 3.9)
Season										
Dry^c^	1664 (37.7)	3.4 (2.5, 4.3)	323 (40.8)	4.6 (2.3, 6.9)	383 (40.5)	5.7 (3.4, 8.1)	603 (42.6)	2.3 (1.1, 3.5)	355 (28.1)	1.7 (0.3, 3.0)
Wet^d^	2753 (62.3)	4.0 (3.3, 4.8)	468 (59.2)	7.7 (5.3, 10.1)	562 (59.5)	3.4 (1.9, 4.9)	814 (57.4)	2.8 (1.7, 4.0)	909 (71.9)	3.6 (2.4, 4.8)
Total	4417 (100)	3.8 (3.2, 4.4)	791 (100)	6.4 (4.7, 8.2)	945 (100)	4.3 (3.0, 5.6)	1417 (100)	2.6 (1.8, 3.4)	1264 (100)	3.1 (2.1, 4.0)
Method of data collection by season										
Web survey, dry^c^ season	425 (9.6)	3.3 (2.8, 3.8)	34 (4.3)	5.9 (0.0, 13.1)	109 (11.5)	6.4 (2.3, 10.6)	272 (19.2)	1.8 (0.5, 3.2)	10 (0.8)	0.0 (0.0, 0.0)
Web-survey, wet^d^ season	780 (17.7)	3.3 (3.0, 3.7)	11 (1.4)	0.0 (0.0, 0.0)	1 (0.1)	0.0 (0.0, 0.0)	335 (23.6)	1.2 (0.2, 2.2)	433 (34.2)	5.1 (3.3, 6.9)
Face-to-face, dry^c^ season	1239 (28.0)	3.5 (3.2, 3.8)	289 (36.5)	4.5 (2.3, 6.7)	274 (29.0)	5.5 (3.0, 5.9)	331 (23.4)	2.7 (1.2, 4.2)	345 (27.3)	1.7 (0.5, 2.9)
Face-to-face, wet^d^ season	1973 (44.7)	4.3 (4.0, 4.6)	457 (57.8)	7.9 (5.6, 10.1)	561 (59.4)	3.4 (2.0, 4.7)	479 (33.8)	4.0 (2.5, 5.4)	476 37.7	2.3 (1.1, 3.5)
Total	4417 (100)	3.8 (3.2, 4.4)	791 (100)	6.4 (4.7, 8.2)	945 (100)	4.3 (3.0, 5.6)	1417 (100)	2.6 (1.8, 3.4)	1264 (100)	3.1 (2.1, 4.0)

a CI (Confidence Interval).

b Since there was no significant difference in AGI risk between the second and third quintiles, and since these households share similar socio-economic conditions, we merged these categories to improve our statistical power to detect wealth-related differences in AGI risk.

c Dry season (Ethiopia: October 1–May 31; Mozambique: April 1–September 30; Nigeria: November 1–March 31; Tanzania: June 1–October 31).

d Wet season (Ethiopia: June 1–September 30; Mozambique: October 1–March 31; Nigeria: April 1–October 31; Tanzania: November 1–May 31).

For those reporting diarrhoea or vomiting in the 4-week recall period, the survey also asked about symptoms; the most loose stools or number of times they vomited on their worst day; if they were sick on the day of data collection; other conditions that could have caused the diarrhoea or vomiting; any care seeking or overnight hospital admissions for the symptoms; if they were asked to provide a stool sample and if they did so; the result of any tests performed on the stool sample (if known); whether they took any medications for their symptoms and the type of medication; and the number of other people in their household who also had diarrhoea or vomiting within the past 4 weeks. For respondents with more than one diarrhoea or vomiting episodes in the 4 weeks before the survey, episodes separated by a 7-day gap were considered separate episodes (and those separated by 6 days or less to be part of the same episode) [26]. Information about handwashing practice (with detergent/disinfectant solution and rubbing hands together for 20 s) and handwashing frequency (average number of times per day within the previous 2 weeks) were also collected, with these variables added in February 2021.

The survey tool was pretested in all six languages with a convenience sample in each of the four countries until no new changes were noted. Pretesting clarified unclear language and fixed errors in skip patterns, and ensured answers were consistent across the six survey languages.

### Data collection

The survey was administered via face-to-face interviews with enumerators and a self-completed web survey to accommodate the lack of internet/technology access and variations in literacy in some study communities. Both modes of administration used the online Qualtrics Insight Platform (Qualtrics, Provo, UT, USA) [21]. For face-to-face interviews, enumerators were trained on and used a prepared tablet. Enumerators used simple random sampling to identify households, then randomly selected a person (with the most recent birthdate or by lottery method) in the house. When the selected individual was unavailable, enumerators either conducted household revisits or picked the following eligible individual. If the selected individual refused to participate, enumerators moved to the next household.

For the web survey, convenience sampling was employed, inviting the study community to participate via a one-page flyer (Supplementary Material). Participants then completed the online survey using an anonymous web link. The survey was promoted via health extension workers, local community gatherings, social media (e.g., WhatsApp), flyers, and posters. Since it was possible to complete the web survey more than once, we included questions about previous participation and timing relative to current participation (e.g., in the last month) to identify repeat responders. Although multiple household members could complete the web survey, no attempt was made to link respondents within households. In countries that offered remuneration, respondents were only remunerated for their first web survey. The web survey was guided through questions, including consent/assent according to the respondent’s age.

Data collection started in February/March 2020 but was paused in all countries on 26 March 2020 due to the COVID-19 pandemic. Data collection resumed in all countries at the beginning of October 2020. Data collected in February/March 2020 and October 2020 were used as the survey pilot. Following pilot data analysis, the survey tool required no changes, and thus the data collected for October 2020 formed the first of the 12-month dataset. Country-specific prevalence estimates from the pilot were less than 10%. Based on the piloting, the target sample size for Mozambique was decreased.

### Analysis

Data for all study communities were analyzed in a single dataset, in SAS 9.4 (SAS Institute, Cary, NC), with incidence rates and proportions calculated using R version 4.0.2. Individuals with ‘do not know/not sure’ responses, who refused to answer a question or provided implausible responses (identified as nonsensical values using population-level information in each country period [5–8], e.g., 60 people sleeping per room, age of 170 years), were excluded from the analysis of that question. Repeat survey participation at any time, and in the previous month, was determined for web survey participants.

AGI was defined using a published standard case definition (≥3 loose stools, or any vomiting, in 24 h, excluding those with cancer of the bowel, irritable bowel syndrome, Crohn’s disease, ulcerative colitis, cystic fibrosis, coeliac disease, or another chronic illness with symptoms of diarrhoea or vomiting; or who report their symptoms were due to drugs, alcohol, or pregnancy) [27]. The DHS definition (diarrhoea in children under 5 years of age during the 2 weeks preceding the survey; number of loose stools not specified) was also applied using children under five with any diarrhoea regardless of the number of stools in a 24 h period [5–8].

AGI incidence rates, proportions, and prevalences were calculated using the formulae and methods in the Supplementary Material. Age standardization of the annual incidence rates was performed using the Nigerian census population age proportion [28], given its larger population than the other study countries. Weighted incidence rates and prevalence were calculated by applying sample weights created for age, gender, and urban/rural status, using the recent census reports in the study countries [28–31].

The mean and median duration of AGI illness were calculated using the time reported by respondents, including those with symptoms still ongoing on the date of data collection. Severity, symptoms, and health-seeking behaviour related to the AGI cases were summarized descriptively. Underreporting was assessed by calculating the ratio of the number of respondents who tested positive (or were asked to submit a sample) to the number with AGI at other steps in the disease surveillance pyramid [32].

All demographic, temporal, and spatial distributions were summarized descriptively. We calculated the 95% confidence interval (CI) for these measures based on the standard formula: 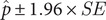 , where 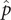 is the observed proportion from our sample, and SE is the standard error, computed as 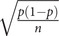 to adjusting for variability based on the sample size. The association between demographic factors and the presence of AGI in the previous 4 weeks was tested, using the χ^2^ test for single factors, and by fitting five multivariable logistic regression models containing all factors (one overall and four country-specific). Individual-level (age and gender), household-level (wealth index, employment status, and the number of people sleeping in the household), and country-level (month, season, and urban/rural status) variables were tested. Here, the employment status of the main earner was further categorized into ‘not working’ (retired, students, housewives, disabled and others) and ‘working’. All demographic variables were included in the final models regardless of their significance to adjust for potential confounding. A two-way interaction effect of selected variables (age with each of gender, residence, wealth, employment status of the main earner, method of data collection, and season; gender with each of residence, wealth, employment status of the main earner, and season; residence with each of wealth, number of people sleeping per room, and season) on the odds of AGI was tested.

The wealth index was used as a proxy for wealth, following a standard approach in equity analysis [33]. Each wealth variable (household utilities, assets, fuel for cooking, and crowding) was recoded into two categories by grouping the response options to where they are more likely to be found: wealthier versus poorer households (Supplementary Material, Table S2). The wealth index was then determined via principal components analysis (PCA), where variables having frequencies between 5% and 95% were included [34]. As assets could vary by worthiness/price between urban and rural areas, the wealth index for urban and rural settings was determined separately for each country and then merged for further analysis. The first component, obtained from the PCA, was used to categorize individuals into five approximate wealth quintiles, ranging from the lowest to the highest quintile [34].

## Results

Overall, 4454 respondents had complete information on variables that measured diarrhoea or vomiting; 37 (0.8%) reported survey participation in the past month and were thus excluded from the analysis. Of the remaining 4417, most (72%) were face-to-face survey respondents, and more females (61%) participated than males. Other demographic characteristics, and numbers of surveys completed by method and season, are shown (Table 1).

**Figure 1.**
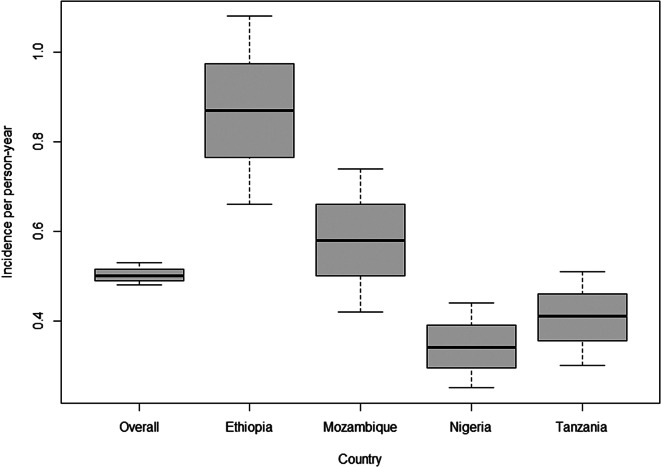
Crude incidence (with the minimum value, the first quartile, the median, the third quartile, the maximum value and 95% confidence interval) of acute gastrointestinal illness in Ethiopia, Mozambique, Nigeria, and Tanzania, and overall, October 2020–September 2021 (n = 4417).

Of the 4417 respondents, 356 (8.0%) had any diarrhoea or vomiting in the 4 weeks prior to the survey, and 168 (3.8%) met the definition for AGI. The crude annual incidence rate was highest in Ethiopia followed by Mozambique (0.87 and 0.58 episodes per person-year, respectively), then Tanzania and Nigeria (0.41 and 0.34 episodes per person-year, respectively) (Figure 1). Rates in Ethiopia and Mozambique increased after age standardization, becoming highest in Mozambique (1.25 and 1.46 episodes per person-year, respectively; Supplementary Table S3), but did not change appreciably in Tanzania or Nigeria (0.58 and 0.33, respectively). The crude incidence proportions and the crude 4-week prevalence followed the same pattern: highest in Ethiopia (0.58 and 6.4%) followed by Mozambique (0.44 and 4.3%), then Tanzania (0.33 and 3.1%) and Nigeria (0.29 and 2.6%), with values increasing for Ethiopia (0.72 and 9.3%) and Mozambique (0.84 and 13.3%) after weighting adjustment (for age, sex, and urban/rural status) resulting in Mozambique having the highest value (Supplementary Table S3). The crude point prevalence was higher in Ethiopia (1.4%) compared to the other three study countries (range: 0.3–0.6), but after weighting adjustment, Mozambique (1.5%) and Ethiopia (1.5%) were comparable.

Of the 4331 respondents with complete information on diarrhoea or vomiting in the past 2 weeks, 56 (1.3%) had AGI in the 2 weeks before the survey. The crude 2-week prevalence of any diarrhoea among children under five was substantially higher in Mozambique (20.8%), followed by Ethiopia (9.0%), compared to Tanzania (1.8%) and Nigeria (0.6%), even after weighting adjustment (16.5% in Mozambique, 9.1% in Ethiopia, 0.8% in Tanzania, and 0.6% in Nigeria; Supplementary Table S3).

More than 86% of the people with AGI in each study country had diarrhoea. Those with AGI had an average of 3.7 to 4.4 stools and 2.3 to 3.2 vomiting episodes on their worst day (Supplementary Table S4). Abdominal pain, stomach pain, fever, headache, and nausea were the most common additional symptoms reported in all countries. The crude mean duration of AGI was 5.3 days in Ethiopia, and 3.0 to 3.4 days elsewhere. At least 30% of people with AGI in all countries had more than one episode in the previous 4 weeks. Among all AGI cases, between 9 % (Tanzania) and 34% (Mozambique) had someone else in the household with AGI.

More than 60% of AGI cases took medication for their symptoms; taking medications was most common in Tanzania (82.1%) and least common in Ethiopia (62.0%; Table 2). In all countries, those with AGI who sought care were more likely to take medications for their symptoms, and the proportion of cases who sought care ranged from 39% (Mozambique) to 74% (Tanzania; Table 2). Of those who sought care, the proportion requested to submit a stool sample ranged dramatically from 69% in Tanzania to 4% in Nigeria. The majority submitted the requested sample, but very few had a positive test result reported to them. The ratio of cases with a positive stool test (or who were asked to submit a sample) to cases of AGI in the community is shown in Table 2.

**Table 2.** Health care-seeking behaviour and medication use among the 168 cases of acute gastrointestinal illness (AGI) in Ethiopia, Mozambique, Nigeria, and Tanzania (October 2020–September 2021)

	Ethiopia		Mozambique		Nigeria		Tanzania	
	Number (%)	Ratio to cases who reported they tested positive	Number (%)	Ratio to cases who submitted a stool sample	Number (%)	Ratio to cases who submitted a stool sample	Number (%)	Ratio to cases who reported they tested positive
Care-seeking (surveillance pyramid steps)								
Cases who reported they tested positive	1 (2.0)	1					9 (23.1)	1
Cases who submitted a stool sample	7 (13.7)	7	1 (2.4)	1	1 (2.7)	1	20 (51.3)	2.2
Cases requested to submit a stool sample for testing	8 (15.7)	8	2 (4.9)	2	1 (2.7)	1	20 (51.3)	2.2
Cases who sought medical care^a^	30 (58.8)	30	16 (39.0)	16	22 (59.5)	22	29 (74.4)	3.2
AGI cases in the community	51 (6.4)	51	41 (4.3)	41	37 (2.6)	37	39 (3.1)	4.3
Medication use								
Cases who took medication for their AGI^b^	31 (60.8)	–	26 (63.4)	–	29 (78.4)	–	32 (82.0)	–
Cases who sought medical care and took medication	28 (54.9)	–	14 (34.1)	–	21 (56.8)	–	29 (74.4)	–

a Medical care includes consultation with a general practitioner, after-hours doctors, pharmacist, or Healthline; visit a private clinic, hospital emergency department, health centre, or nursing services; or use of alternative health care (e.g., naturopathy, homeopathy, chiropractic, or herbalist).

b Medication they took includes medicine to stop diarrhoea (e.g., immodium, lomotil); medicine to prevent nausea (e.g., maxalon, stemetil); antibiotics (e.g., amoxil, synermox, erythromycin, bactrim); or others (e.g., Metronidazole, Paracetamol, medicine to stop malaria, traditional drug, Azithromycin).

**Figure 2.**
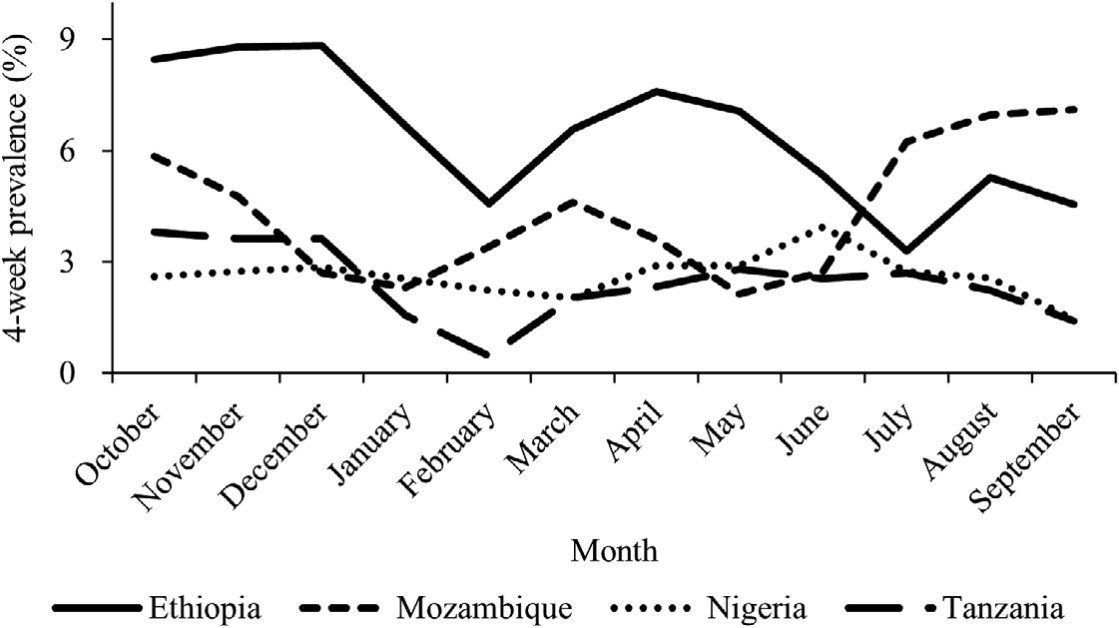
Three-month moving average of the crude 4-week prevalence of acute gastrointestinal illness in Ethiopia, Mozambique, Nigeria, and Tanzania, October 2020–September 2021 (n = 4417).

The 4-week prevalence varied monthly; most notable was the higher value in September compared to July in Mozambique (Figure 2). While the odds of having AGI were not significantly associated with wet or dry seasons in any of the countries (Table 3), the overall pooled odds ratio (OR = 1.25, 95% CI: 0.88, 1.76) and the ORs in Ethiopia (OR = 1.59, 95% CI: 0.82, 1.76), Nigeria (OR = 1.23, 95% CI: 0.59, 2.57), and Tanzania (OR = 1.60, 95% CI: 0.59, 4.35) were greater than one, suggesting a possible modest increased risk during the wet seasons. In Mozambique, however, the OR was less than one (OR = 0.54, 95% CI: 0.25, 1.14). However, the proportion of surveys completed via the web during the wet season was relatively low in Mozambique (11/468; 2.4%) and Ethiopia (1/562; 0.18%), compared to Nigeria (335/814; 41.2%) and Tanzania (443/909; 47.6%; Table 1). Additionally, individuals who responded via the web survey were two times more likely to have AGI than individuals who responded face-to-face (95% CI: 1.29–3.48; Table 3), suggesting that the lack of seasonal association observed here may be due to differences in survey method abilities to measure AGI occurrence.

**Table 3.** Odds of having acute gastrointestinal illness (AGI) by demographic characteristic in Ethiopia, Mozambique, Nigeria, and Tanzania, adjusted for all variables in the models, October 2020–September 2021 (n = 4417), significant values at α = 0.05 are in bold

Variable	Overall		Ethiopia		Mozambique		Nigeria		Tanzania	
	Odds Ratio	95% Confidence Interval	Odds Ratio	95% Confidence Interval	Odds Ratio	95% Confidence Interval	Odds Ratio	95% Confidence Interval	Odds Ratio	95% Confidence Interval
Gender										
Male	1.11	0.79, 1.56	1.33	0.68, 2.58	0.75	0.36, 1.56	1.23	0.61, 2.48	1.00	0.49, 2.03
Female	Ref.	Ref.	Ref.	Ref.	Ref.	Ref.	Ref.	Ref.	Ref.	Ref.
Age (years)										
0–4	**4.36**	**2.85, 6.66**	**4.14**	**2.16, 7.94**	**4.10**	**1.08, 15.57**	**3.24**	**1.37, 7.66**	**6.89**	**2.55, 18.62**
5–14	**2.24**	**1.13, 4.42**	4.92	0.98, 24.75	**13.06**	**3.01, 56.69**	1.49	0.48, 4.65	0.65	0.08, 5.12
15–59	Ref.	Ref.	Ref.	Ref.	Ref.	Ref.	Ref.	Ref.	Ref.	Ref.
60+	1.04	0.55, 1.97	0.95	0.32, 2.85	2.48	0.83, 7.43	2.21	0.22, 22.04	0.51	0.11, 2.41
Wealth index quintile										
Lowest	1.38	0.88, 2.17	1.00	0.40, 2.54	1.19	0.51, 2.75	2.97	0.95, 9.34	1.72	0.64,4.57
Second & Middle	Ref.	Ref.	Ref.	Ref.	Ref.	Ref.	Ref.	Ref.	Ref.	Ref.
Fourth	**1.68**	**1.10, 2.57**	1.83	0.87, 3.87	0.66	0.25, 1.73	**4.57**	**1.55, 13.45**	2.45	0.97, 6.19
Highest	1.16	0.72, 1.84	1.06	0.46, 2.43	0.51	0.18, 1.42	**4.02**	**1.33, 12.12**	1.19	0.39, 3.67
Residence										
Urban	Ref.	Ref.	Ref.	Ref.	Ref.	Ref.	Ref.	Ref.	Ref.	Ref.
Rural	**1.56**	**1.08, 2.26**	0.91	0.50, 1.69	1.50	0.70, 3.22	1.91	0.63, 5.83	1.96	0.95, 4.04
Employment status of the main earner in the household										
Working	1.26	0.83, 1.92	1.21	0.59, 2.50	**3.74**	**1.31, 10.65**	1.13	0.33, 3.87	0.76	0.33, 1.75
Not working	Ref.	Ref.	Ref.	Ref.	Ref.	Ref.	Ref.	Ref.	Ref.	Ref.
No. people sleeping per room										
≤ 3	Ref.	Ref.	Ref.	Ref.	Ref.	Ref.	Ref.	Ref.	Ref.	Ref.
> 3	1.29	0.85, 1.95	1.20	0.60, 2.40	1.03	0.37, 2.91	1.07	0.49, 2.32	1.18	0.42, 3.36
Method of data collection^a^										
Web survey	**2.12**	**1.29, 3.48**	0.90	0.20, 4.15	2.11	0.65, 6.88	–	–	2.55	0.98, 6.63
Face-to-face	Ref.	Ref.	Ref.	Ref.	Ref.	Ref.	–	–	Ref.	Ref.
Season										
Dry^b^	Ref.	Ref.	Ref.	Ref.	Ref.	Ref.	Ref.	Ref.	Ref.	Ref.
Wet^c^	1.25	0.88, 1.76	1.59	0.82, 3.09	0.54	0.25, 1.14	1.23	0.59, 2.57	1.60	0.59, 4.35
Country										
Nigeria	Ref.	Ref.	–	–	–	–	–	–	–	–
Ethiopia	**3.91**	**2.30, 6.66**	–	–	–	–	–	–	–	–
Mozambique	**3.84**	**2.24, 6.60**	–	–	–	–	–	–	–	–
Tanzania	**2.11**	**1.25, 3.55**	–	–	–	–	–	–	–	–

a Method of data collection was removed from the final model for Nigeria because of its similarity with the variable residence.

b Dry season (Ethiopia: October 1–May 31; Mozambique: April 1–September 30; Nigeria: November 1–March 31; Tanzania: June 1–October 31).

c Wet season (Ethiopia: June 1–September 30; Mozambique: October 1–March 31; Nigeria: April 1–October 31; Tanzania: November 1–May 31).

Age was the only factor significantly associated with having AGI in all four countries, with higher odds in those 0–4 compared to those 15–59 years (overall OR 4.3, 95% CI: 2.85, 6.66; Table 3), ranging from 3.2 times higher in Nigeria, to 6.9 times higher in Tanzania. Overall, children 5–14 years also had higher odds of AGI, though this was only significant in Mozambique (OR = 13.06; 95% CI: 3.01, 56.69), while Tanzania (OR = 0.65; 95% CI: 0.08, 5.12) showed lower odds. Among adults 60+ years, the odds ratios were highly variable across countries, with no clear trend. While the OR in Mozambique (OR = 2.48) may indicate potential increased odds of AGI in older adults, other countries had much smaller or inconsistent effects (e.g., Ethiopia: OR = 0.95, 95% CI: 0.32, 2.85; Tanzania: OR = 0.51, 95% CI: 0.11, 2.41).

The employment status of the household’s main earner was most strongly associated with AGI in Mozambique, where individuals from a household with working main earners were 3.7 times more likely to have AGI than households without working main earners (95% CI: 1.31–10.65); other countries did not show a strong association, as all had non-significant odds ratios close to 1 (Table 3). The relationship between wealth index and AGI varied by country, and was only significant in Nigeria, where individuals in a household in the fourth (OR = 4.57, 95% CI: 1.55–13.45) and highest-level (OR = 4.02, 95% CI: 1.33–12.12) assets wealth quintiles were four times more likely than those with second-and middle-level assets to have AGI (Table 3). In Ethiopia and Tanzania, the highest odds of AGI was also for individuals in a household in the fourth wealth quintile, whereas the highest odds of AGI in Mozambique was for individuals in the lowest wealth quintile, though all had non-significant odds ratios (Table 3).When all countries were analyzed together, adjusted for all other factors in the model including country, the only variable that changed universally was urban/rural status, which changed from non-significant in all four country models to significant. Specifically, individuals from rural sites were 1.56 times more likely than those from urban areas to have AGI (95% CI: 1.08, 2.26; Table 3). No other changes to the magnitude, sign, significance, general trends were observed. Neither handwashing (OR = 1.14, 95% CI: 0.67–1.93) nor handwashing frequency (OR = 0.98, 95% CI: 0.95–1.01) were strongly or significantly associated with the odds of AGI after adjusting for the other variables (Supplementary Material, Tables S5–S7).

## Discussion

This study aimed to describe the epidemiology of AGI for individuals of all ages in Ethiopia, Mozambique, Nigeria, and Tanzania. Previous studies on AGI or diarrhoea in these countries have almost exclusively focused on under-five children (e.g., [9–13]). Thus, ours is the first primary study to measure the incidence, prevalence and duration of AGI, and identify demographic determinants, across all ages in these four countries, using standardized methods and tools and an internationally accepted case definition to facilitate comparisons [27]. Our findings confirmed that while the epidemiology of AGI in the general population in Ethiopia, Mozambique, Nigeria and Tanzania is largely comparable to what has been observed across HICs and LMICs, and that children under five remain a risk group, some important differences exist.

Overall, annual incidence rates in this study were comparable or slightly lower than those reported for other African and Caribbean LMICs (e.g., [22, 23, 35–37]), as well as Asian and Latin and Caribbean LMICs from studies using similar case definitions (e.g., [38–40]). However, these studies were done before the COVID-19 pandemic, so it is possible that our AGI estimates were lower because of the extensive promotion of handwashing practices, including the use of alcohol or sanitizer to clean hands and recommendation for social distancing during the study period [41]. Unfortunately, although we collected handwashing-related data, we did not observe a significant association between handwashing and lower odds of AGI.

Between our four countries, the annual incidence of AGI was higher in Ethiopia and Mozambique compared to Nigeria and Tanzania, even after weighting and age standardization. Indeed, the annual incidences in Nigeria and Tanzania were more comparable to some higher-income settings [40, 42, 43]. Nigeria and Tanzania are currently both lower-middle-income countries, whereas Ethiopia and Mozambique are low-income countries [44], so lower rates of AGI may be due to improved water sources, toilet facilities, and waste disposal methods [45, 46], particularly since previous studies in these four countries identified unimproved water, sanitation, and hygiene as risk factors for diarrhoeal illness (e.g., [12, 13, 47, 48]).

In Ethiopia, Nigeria, and Tanzania, children under five had the highest prevalence of AGI, and the highest odds of AGI even after adjusting for urban/rural status, gender, wealth, occupation, season, and the size of the household. This was expected and is consistent with studies in other LMICs [20, 37–40, 49]. In Mozambique, however, children aged 5–14 years were at the same risk as children under five, with the highest prevalence and odds although confidence intervals overlapped. This finding may be spurious, as there were few participants aged 5–14 years. It may however reflect an actual increase in risk, for example due to cholera which surged in Mozambique in 2021 [50, 51].

To compare our results to those from the DHS survey, we applied their case definition and calculated the two-week prevalence of diarrhoea in children under five [5–8]. Except for Ethiopia, where our estimate (9% in 2020–2021) was comparable to the most recent DHS estimate (12% in 2016; [5]), our estimates were vastly different than the most recent DHS estimates available. In Mozambique, our estimate for 2020–2021 (21%) was twice as high as the DHS estimate (9% in 2022/2023) a year later [6]), again potentially due to the surge in cholera cases during our study period [50, 51]. In Nigeria and Tanzania, our estimates (0.6% and 1.8%, respectively) were roughly ten times smaller than the most recent DHS estimates (13% in 2018 [7]; 9% in 2022 [8]). In Nigeria, our more current estimate may be lower due to the recent, extensive provision of zinc and oral rehydration solutions to children with diarrhoea, reducing subsequent diarrhoeal episodes [52–54], or to improve hand hygiene of children and caretakers to prevent COVID-19 [55]. In Tanzania, the reasons are less clear. National DHS estimates in Tanzania have declined since 2015/16 (12%; [56]), likely due to the scaling up of childhood interventions like improved nutrition and vaccination [57]. However, the 10-fold difference in estimates in the same time period may be due to study design differences. While DHS surveys use a country-wide multi-stage sampling scheme to capture all population segments, surveys only capture a 4- to 7-month timeframe [5–8]. In contrast, our study only included one urban and one rural site per country, but captured a 12-month timeframe to account for temporal variations in prevalence, as was observed in Tanzania. The impact of these methodological differences on estimated AGI prevalence bears further investigation.

The mean duration of illness and average maximum number of loose stools and vomiting episodes in 24 h in our four study countries is comparable to what has been reported in other studies conducted before the COVID-19 pandemic, from both HICs [16, 18, 42, 58, 59] and LMICs [20, 35, 37–40, 49, 60]. While indicators of severity were generally consistent across our four countries, overall severity appeared somewhat worse in Ethiopia, where those with AGI had on average the longest duration of illness (2 days longer than in Mozambique, Nigeria, and Tanzania), experienced on average more times vomiting or loose stools on their worst day, and more fever and headaches. More prolonged diarrhoeal episodes occur in younger ages [61], and Ethiopia had the highest relative number of under-five children with AGI compared to the other three study countries; however, even after adjusting the mean duration for age, sex, and urban/rural status, Ethiopia had the longest average duration by at least a day. It is possible that the higher prevalence of malnutrition among under-fives in Ethiopia, compared with the other three study countries [62], contributed to the longer relative duration [63]. Here, AGI was least severe in Mozambique, where those with AGI had on average the shortest duration of illness, and the fewest average times vomiting or loose stools on their worst day. In light of the higher incidence observed in Mozambique, together with the surge in cholera that occurred [50, 51], this lower severity may indicate milder cholera cases due to improved education about case management, or vaccination eliciting less of an immune response in populations where cholera is endemic [64, 65].

Consistent with observations about severity, seeking care for AGI was also less frequent in Mozambique than in the other four countries. While severity of illness may drive health care-seeking behaviours, access to care is also important. Tanzania has a slightly lower average travel time to access the nearest public healthcare facility compared to Ethiopia and Mozambique [66]. Additionally, in Ethiopia and Nigeria, a significant proportion of people could not receive care from 2020 to 2022 due to fear of catching COVID-19, inadequate supplies or tests at the hospital or clinic, or closure of the medical facility [67]. These may be reasons for the differences observed here.

In many countries, AGI has a seasonal pattern (e.g., [20, 49, 60, 68]). Here, while the 4-week prevalence did vary across study months in Ethiopia, Mozambique, and Tanzania, the incidence of AGI did not vary significantly between each country’s wet and dry seasons, including after adjusting for other factors like method of data collection. The small number of AGI cases (n = 168) may have made it difficult to detect true seasonality. It is also possible that improved hand hygiene to prevent COVID-19 [69] impacted seasonal patterns during our study timeframe. However, it is more likely that the lack of web surveys in Mozambique and Ethiopia during their wet seasons, combined with our finding that those completing web surveys had a higher odds of AGI than those completing face-to-face surveys, created a differential selection bias by survey method that masked the true relationship between AGI and season. Future studies should explore this further.

In this study, we assessed whether household main earner status (working or not) and wealth index quintile were associated with AGI, since main earner status and related economic activities and perception of symptoms may relate to both real and reported AGI prevalence [38, 39, 68], and since wealth quintiles are linked to things like unimproved water supply and sanitation (lowest quintiles) and eating out (higher quintiles) which affect AGI occurrence [20, 38, 70, 71]. While these factors were not universally associated with AGI across the four countries, some notable patterns emerged in specific settings. In Mozambique, individuals in households with a working main earner had significantly higher odds of AGI compared to those in households where the main earner was retired, disabled, a student or housewife, and others. The large effect size suggests that economic activity may influence exposure to AGI risk factors, possibly through workplace or travel-related transmission, though the confidence interval indicates some uncertainty in this estimate. Wealth index was only significantly associated with AGI in Nigeria, where individuals in households in the fourth- and highest-level wealth quintiles were four times more likely to experience AGI than those in households in the second- and middle-level wealth quintiles. This suggests that wealthier households may experience AGI due to lifestyle-related factors such as increased eating out or travel; however, wide confidence intervals indicate uncertainty in the estimate. Nigeria and Tanzania are both lower-middle income countries, whereas Ethiopia and Mozambique are low-income [44], so we expected potential consistency between these pairs of countries. Unfortunately, it is difficult to determine if the results observed here reflect reporting bias (working or wealthier households more likely to report AGI), factors such as lifestyle (e.g., travel or eating out; [38]), or an insufficient sample size in some countries. Ways in which the main earner’s status and wealth influence the risk of AGI in these countries need further investigation.

For practical reasons, in part due to the COVID-19 pandemic, we used both face-to-face and web survey methods. Individuals who completed the web survey had a higher odds of AGI than those who participated in the face-to-face survey, adjusting for other factors such as urban/rural status and wealth. It is possible that the web survey format allowed participants to report AGI symptoms more comfortably than the face-to-face format involving an enumerator [72]. It is also possible that web survey respondents differed in ways we did not measure, such as education, and were more prone to reporting symptoms due to better access to the internet/technology, awareness, or concern.

This study is subject to several limitations, common to population surveys of AGI [20, 49]. Recall bias and misclassification bias are possible given self-reported symptoms [73]. To minimize this, we conducted extensive training of survey enumerators, including on optimal times for in-person data collection, and used control logic in the online survey to reduce mistyping [73]. Response and social desirability bias are also limitations of face-to-face surveys [74]. Here, data collectors interviewed privately where possible, to reduce the influence of family or other bystanders during the survey [74], and allowed participants to think before responding, kept reactions to participants’ responses neutral, and assured the confidentiality of the information participants gave [75]. Additional bias mitigation strategies were used in survey design, for example avoiding leading and loaded questions, and offering the response option ‘prefer not to respond’ for each question [76]. Finally, given our sample size, we were unable to perform extensive sub-group analyses, for example, AGI occurrence based on access to health care, where future studies can use a prospective cohort design to investigate such issues further.

In conclusion, this study provides the first measures of the incidence, prevalence, duration and demographic distribution of AGI, for all ages, in Ethiopia, Mozambique, Nigeria, and Tanzania, using standardized methods and tools and an internationally accepted case definition. Overall, our observations of AGI in the population generally compare to what has been observed in similar studies in other countries, suggesting that the epidemiology of AGI is shifting, perhaps in response to interventions and prevention measures implemented over previous years.

## Supporting information

Desta et al. supplementary material Desta et al. supplementary material

## Data Availability

The data (with SAS and R code) that support the findings of this study will be made openly available in the Foodborne Disease Epidemiology, Surveillance, and Control in African LMIC (FOCAL) Population Survey (2021–2022) folder within the University of Waterloo Dataverse Collection at https://borealisdata.ca/dataverse/focal-population-survey.

## Supplementary material

For supplementary material accompanying this paper visit http://doi.org/10.1017/S095026882500038X. click here to view supplementary material
