# Supplementary material for: The epidemiology of acute gastrointestinal illness in Ethiopia, Mozambique, Nigeria, and Tanzania: a population survey
Source: Epidemiol Infect. 2025 Apr 21;153:e63. doi: 10.1017/S095026882500038X (PMC12086602; doi:10.1017/S095026882500038X)
Supplement: Desta et al. supplementary material [file S095026882500038Xsup001.docx]

**Epidemiology and Infection**

**The epidemiology of acute gastrointestinal illness in Ethiopia, Mozambique, Nigeria, and Tanzania: a population survey**

Binyam N. Desta, Sara M. Pires, Tine Hald, Tesfaye Gobena, Custodia Macuamule, Belisario Moiane, Olanrewaju E. Fayemi, Christianah I. Ayolabi, Gabriel Akanni, Blandina T. Mmbaga, Kate M. Thomas, Happiness Kumburu, Warren Dodd, and Shannon E. Majowicz

**Supplementary Materials**

**Supplementary Table S1.** Summary of details about each of the study sites of a cross-sectional survey of acute gastrointestinal illness in Ethiopia, Mozambique, Nigeria, and Tanzania, October 2020 – September 2021

|  | | Ethiopia | | Mozambique | | Nigeria | | Tanzania | | |
| --- | --- | --- | --- | --- | --- | --- | --- | --- | --- | --- |
|  |  | **Urban** (Harar) | **Rural**  (Kersa) | **Urban**  (Polana Caniço -KaMaxaque) | **Rural**  (Marracuene) | **Urban**  (Abeokuta, Ogun State) | **Rural**  (Ofada/ Mokoloki, Local Council Development Area, Ogun State) | **Urban**  (Moshi Municipal) | | **Rural**  (Moshi Rural) |
| Population of study site | | 99,321 | 172,626 | 199,565 | 230,530 | 557,000 | 230,000 | 184,292 | | 466,737 |
| Initial target sample size | | 372 | 372 | 984 | 984 | 552 | 552 | 432 | | 408 |
| Revised target sample size, post-pilot | | 372 | 372 | 552 | 552 | 552 | 552 | 432 | | 408 |
| Initial start date of data collection | | March 11, 2020 | | March 18, 2020 | | February 21, 2020 | | February 17, 2020 | | |
| Data collection pause date (due to the COVID-19 pandemic) | | March 26, 2020 | | March 26, 2020 | | March 26, 2020 | | March 26, 2020 | | |
| Data collection resume date | | May 31, 2020 | | September 16, 2020 | | June 03, 2020 | | October 09, 2020 | | |
| Pilot study | Duration | March 11, 2020, to October 31, 2020 | | March 18, 2020, to October 31, 2020 | | February 21, 2020, to October 31, 2020 | | February 27, 2020, to October 31, 2020 | | |
|  | Sample size used | 117 | 70 | 187 | 108 | 117 | 185 | 55 | 74 | |
| Main study | Duration | October 01, 2020, to September 30, 2021 | | October 01, 2020, to September 30, 2021 | | October 01, 2020, to September 30, 2021 | | October 09, 2020, to September 30, 2021 | | |
|  | Sample size used | 409 | 383 | 428 | 520 | 660 | 759 | 749 | | 546 |
| No of people surveyed more than once | With in a month | 1 | | 3 | | 2 | | 31 | | |
|  | Total | 5 | | 22 | | 7 | | 148 | | |
| Format of survey administration | | Web and face-to-face | Web and face-to-face | Web and face-to-face | Web and face-to-face | Web and face-to-face | Face-to-face only | Web and face-to-face | | Web and face-to-face |
| Survey languages | | Amharic^1^, Afaan Oromoo^2^ | | Portuguese^3^ | | Yoruba^4^, English^5,6^ | | Kiswahili^7^ | | |
| Ethical review boards | Local | Institutional Health Research Ethics Review Committee (IHRERC) of Haramaya University (#Not provided); National Research Ethics Committee (#MoSHE//RD/14.2/9849/12) | | The National Bioethical committee, Ministry of Health (#CIBS FM&HCM/092/2019) | | Federal Medical Centres (#NHREC/08/10-2015); State Health Service Commission (#02/10/19/041); State Hospital Management Board (#SHH/EC/EA/03/07/20) | | Kilimanjaro Christian Medical Centre (KCMC) (#2446); National Institute of Medical Research (NIMR) (#NIMR/HQ/R.8a/Vol. IX/3273) | | |
|  | International | A University of Waterloo Research Ethics Committee (ORE #40458; Canada) | | | | | | | | |
| Age of assent to participate, and to complete the survey themselves (years) | | 14-17 | | 12-17 | | 14-17 | | 14-17 | | |
| Remuneration | | None | | 1USD per online survey, for internet data | | None | | 1USD per online survey, for internet data | | |

^1^ Gobena, Tesfaye; Desta, Binyam N.; Hailu, Yonas, 2024, "FOCAL Population Survey Tool (Amharic translation)", <https://doi.org/10.5683/SP3/ERXYAM> , Borealis, V1

^2^ Gobena, Tesfaye; Girma, Sagni; Adare, Dechasa; Belina, Dinaol; Tabessa, Nabek, 2024, "FOCAL Population Survey Tool (Afaan Oromoo translation)", <https://doi.org/10.5683/SP3/UDKPNZ> , Borealis, V1

^3^ Macuamule, Custódia; Salvador, Elsa Maria; Moiane, Belisário Tomé, 2024, "FOCAL Population Survey Tool (Portuguese translation)", <https://doi.org/10.5683/SP3/RFN9HN> , Borealis, V1

^4^ Fayemi, Olanrewaju E.; Ayolabi, Christianah I.; Akanni, Gabriel Bidemi, 2024, "FOCAL Population Survey Tool (Yoruba translation)", <https://doi.org/10.5683/SP3/TW3XIM> , Borealis, V1

^5^ Desta, Binyam N.; Hald, Tine; Pires, Sara M.; Gobena, Tesfaye; Macuamule, Custódia; Fayemi, Olanrewaju E.; Ayolabi, Christianah I.; Mmbaga, Blandina T.; Thomas, Kate M.; Dodd, Warren; Hailu, Yonas; Salvador, Elsa Maria; Moiane, Belisário Tomé; Akanni, Gabriel Bidemi; Kumburu, Happiness; Njage, Patrick Murigu Kamau; Colverson, Kathleen Earl; Majowicz, Shannon E., 2024, "FOCAL Population Survey Tool (English): A survey to determine the epidemiology of acute gastrointestinal illness in Ethiopia, Mozambique, Nigeria, and Tanzania", <https://doi.org/10.5683/SP3/GUIIHG> , Borealis, V1

^6^ Desta, Binyam N.; Majowicz, Shannon E., 2023, "Appearance of the ‘FOCAL Population Survey Tool (English)’ in Qualtrics", <https://doi.org/10.5683/SP3/IGLSQ8> , Borealis, V1

^7^ Mmbaga, Blandina T.; Kumburu, Happiness; Hugho, Ephrasia, 2024, "FOCAL Population Survey Tool (Swahili translation)", <https://doi.org/10.5683/SP3/F9DJKX> , Borealis, V1

**One pager-flyer**

**YOUR PARTICIPATION IS NEEDED!**

**Participate in a research study!**

***Do you know?***

In Africa, 1 in 10 child deaths and 1 in 13 all-ages deaths are due to diarrheal illnesses, and majority occurs in sub-Saharan Africa including Ethiopia, Mozambique, Nigeria, and Tanzania. These illnesses continue to be a concern, even during the current COVID 19 pandemic.

Participation in the study will not necessarily lead to controlling diarrheal disease.

Please use your smartphone or computer, open your internet browser and type (**the web-address**) and complete the survey which might take 10 to 15 minutes of your time!

The webpage will be active starting from (**Month year**)

By doing so, you will be part of the effort aiming to contribute to the reduction of illness and death from diarrheal disease, and at the same time, you will be reimbursed for your airtime.

This survey study is planned to estimate the occurrence and distribution of diarrheal disease, and its risk factors in Ethiopia/Mozambique/Nigeria/Tanzania. The study finding will be used to estimate the number of people suffering from diarrheal illness, thereby facilitating disease control by giving information on who needs the resource the most. We will be able to see how the risk changes over time, including as wider factors like the COVID-19 pandemic change.

This study has been reviewed by, and received ethics clearance through a University of Waterloo Research and <<COUNTRY/ INSTITUTION>> Ethics Committees.

__________University in collaboration with the Technical University of Denmark, the University of Waterloo (Canada), and partner Universities from four African countries lead the survey study.

If you have any inquiries, please call us on (**phone number of local collaborator**) or email to Dr. Shannon Majowicz ([smajowicz@uwaterloo.ca](mailto:smajowicz@uwaterloo.ca)) or Mr. Binyam Desta ([bndesta@uwaterloo.ca](mailto:bndesta@uwaterloo.ca)).

*Your participation and genuine response contribute a lot to your community and the world!*

**Formulae used for Incidence and Prevalence Calculations**

Here we present additional details about the Acute Gastrointestinal Illness (AGI) incidence and prevalence calculation we performed. For respondents reporting more than one episode, the most recent episode was used when calculating incidence (i.e., individuals with more than one episode of AGI in the 4-week period were included in the numerator only once for the incidence calculation). To calculate the incidence, the numerator was the number of respondents with AGI during the 4-week recall. Since this group could include individuals ill during the 4-week recall period but whose symptoms started before the 4-week period, the incidence rate was adjusted to account for the likely proportion of episodes that started before the 4-week period. For this purpose, first, an assumption was made that AGI cases occurred evenly throughout the 4-week period. Then, the average duration of illness was used to estimate the probable proportion of illnesses that began before the 4-week period. The incidence rate was adjusted by subtracting this proportion from the numerator and denominator of the incidence rate calculation. The 4-week prevalence of AGI was calculated as the proportion of respondents with AGI in the 4-week recall period, regardless of the illness start date. A similar calculation was done to determine both a 2-week prevalence of AGI in all respondents, and a 2-week prevalence of diarrhoea among children under five years (i.e., the DHS definition). The point prevalence was calculated as the proportion of respondents with AGI on the day of the data collection.

***Formulae to adjust for the 4-week incidence rate***

$$\frac{[Average Duration of Illness - 1]}{[28+(Average Duration of Illness - 1)]}$$

***Formulae to calculate the incidence and prevalence rates***

***Annual incidence rate***

*(4-week recall)*

$$= \frac{Incident cases}{\frac{1}{2}[\left( Total population at risk \right)+\left( Total population at risk-Incident cases \right)]}* \frac{365}{28}$$

***Annual incidence proportion***

*(4-week recall)*

$$= {1-(1-x)}^{\frac{365}{28}}$$

Where, x represents number of incident cases divided by total population at risk.

***Period prevalence***

*(4-week recall)*

$$= \frac{Number of cases in the 28 days of recall}{Total population at risk}$$

*(2-week recall)*

$$= \frac{Number of cases in the 14 days of recall}{Total population at risk}$$

References

**Greenland S, Rothman K**. *Measures of Occurrence*. *In: Rothman KJ, Greenland S, Lash TL, Eds. Modern epidemiology, 3rd ed. Philadelphia: Wolters Kluter*. 2008, p. 32–50.

**Thomas MK, *et al.*** Population distribution and burden of acute gastrointestinal illness in British Columbia, Canada. *BMC Public Health* 2006; **6**: 307.

**Supplementary Table S2.** Wealth variables collected in a cross-sectional survey of acute gastrointestinal illness, assigned to where they are likely to be found, wealthier versus poorer, Ethiopia, Mozambique, Nigeria, and Tanzania, October 2020 – September 2021

| Variable | Wealthier | Poorer |
| --- | --- | --- |
| Source of drinking water | 1 = Piped into dwelling/yard or communal tap or neighbors’ home or protected well/spring or water from rain or tanker/trunk or bottled/sachet water | 0 = Unprotected well/spring or surface-river, lake, dam |
| Type of toilet | 1 = Flushed to piped sewer system or flush to septic tank or Ventilated-Improved-Pit latrine or pit latrine with slab or unshared facility | 0 = Pit latrine with no slab or no latrine/open field or shared facility |
| Floor material | 1 = Rudimentary wood plank, adobe, polished wood, ceramic tile/brick, cement | 0 = earth, sand, or dung |
| Wall material | 1 = Masica stick or casca or wood or metal planks or adobe or brick or cement block | 0 = No walls or grass/thatch/mud or tin/cardboard/paper |
| Roof material | 1 = Iron sheet or calamine/cement fiber or tile or cement/concrete | 0 = No roof or grass/thatch/mud |
| Fuel for cooking | 1 = Electricity or kerosene or cooking gas | 0 = Coal/lignite or charcoal or firewood or dung |
| Has an electric supply | 1 = Yes | 0 = No |
| Has a TV | 1 = Yes | 0 = No |
| Has a radio | 1 = Yes | 0 = No |
| Has a refrigerator (functional) | 1 = Yes | 0 = No |
| Has a bicycle | 1 = Yes | 0 = No |
| Has a motor bicycle/scooter | 1 = Yes | 0 = No |
| Has a car/ truck | 1 = Yes | 0 = No |
| Has an animal-drawn cart | 1 = Yes | 0 = No |
| Has a boat with motor | 1 = Yes | 0 = No |
| Has a mobile phone | 1 = Yes | 0 = No |
| Has a watch | 1 = Yes | 0 = No |
| No. people sleeping per room | 1 = Five or fewer | 0 = Six or more people |
| Own an agriculturally usable land | 1 = Yes | 0 = No |
| Have cows/bulls | 1 = Yes | 0 = No |
| Have horses/ donkeys/ mules | 1 = Yes | 0 = No |
| Have goats | 1 = Yes | 0 = No |
| Have sheep | 1 = Yes | 0 = No |
| Have chickens | 1 = Yes | 0 = No |
| Have ducks | 1 = Yes | 0 = No |
| Have pigeons | 1 = Yes | 0 = No |
| Have pigs | 1 = Yes | 0 = No |
| Have a bank account/ or belong to a savings or micro-credit group | 1 = Yes | 0 = No |
| Own a house | 1 = Yes | 0 = No |
| Own a land | 1 = Yes | 0 = No |

**Table S3** Incidence and prevalence of acute gastrointestinal illness (AGI) in Ethiopia, Mozambique, Nigeria, and Tanzania, October 2020 – September 2021 (n=4417)

|  | | | Overall | Ethiopia | Mozambique | Nigeria | Tanzania |
| --- | --- | --- | --- | --- | --- | --- | --- |
| Incidence (per person-year) (95% C.I.) | | | | | | | |
| Annual rate | Crude | | 0.50 (0.48, 0.53) | 0.87 (0.66, 1.08) | 0.58 (0.42, 0.74) | 0.34 (0.25, 0.44) | 0.41 (0.30, 0.51) |
|  | Weighted** | | 0.81 (0.51, 1.08) | 1.26 (0.22, 1.92) | 1.86 (0.65, 2.81) | 0.32 (0.21, 0.42)*** | 0.43 (0.22, 0.60) |
|  | Age-standardized**** | | 0.57 (0.52, 0.62) | 1.25 (0.45, 1.67) | 1.46 (0.19, 2.03) | 0.33 (0.15, 0.49) | 0.58 (0.23, 0.79) |
|  | Crude by gender | Male | 0.53 (0.49, 0.56) | 1.18 (0.70, 1.57) | 0.54 (0.29, 0.76) | 0.39 (0.25, 0.52) | 0.46 (0.30, 0.61) |
|  |  | Female | 0.49 (0.47, 0.52) | 0.78 (0.55, 0.97) | 0.60 (0.41, 0.77) | 0.31 (0.21, 0.42) | 0.36 (0.23, 0.48) |
|  | Crude by age | 0-4 | 1.29 (1.21, 1.37) | 2.31 (1.58, 2.89) | 1.70 (0.00, 2.67) | 0.78 (0.51, 1.01) | 1.79 (0.61, 2.36) |
|  |  | 5-14 | 0.56 (0.48, 0.63) | 2.05 (0.00, 2.99) | 3.65 (0.00, 5.17) | 0.34 (0.07, 0.57) | 0.36 (0.00, 0.62) |
|  |  | 15-59 | 0.38 (0.37, 0.40) | 0.58 (0.38, 0.78) | 0.48 (0.34, 0.62) | 0.18 (0.09, 0.27) | 0.36 (0.24, 0.47) |
|  |  | 60+ | 0.38 (0.33, 0.44) | 0.51 (0.04, 0.90) | 0.68 (0.15, 1.07) | 0.32 (0.00, 0.81) | 0.14 (0.00, 0.26) |
| Annual proportion (95% CI*) | Crude | | 0.40 (0.38, 0.41) | 0.58 (0.48, 0.66) | 0.44 (0.34, 0.52) | 0.29 (0.22, 0.35) | 0.33 (0.26, 0.40) |
|  | Weighted** | | 0.55 (0.40, 0.66) | 0.72 (0.19, 0.85) | 0.84 (0.48, 0.94) | 0.27 (0.19, 0.34)*** | 0.35 (0.20, 0.45) |
| Prevalence | | | | | | | |
| 4-week (all respondents) | Total cases | | 168 | 51 | 41 | 37 | 39 |
|  | Crude (95% CI) | | 3.8 (3.6, 4.0) | 6.4 (4.9, 8.0) | 4.3 (3.2, 5.5) | 2.6 (1.9, 3.3) | 3.1 (2.3, 3.9) |
|  | Weighted (95% CI)** | | 6.0 (3.7, 8.3) | 9.3 (1.7, 16.7) | 13.3 (5.0, 21.7) | 2.4 (1.5, 3.3)*** | 3.2 (1.6, 4.9) |
| 4-week (web-survey respondents only) | Total cases | | 40 | 2 | 7 | 9***** | 22 |
|  | Crude (95% CI) | | 3.3 (3.0, 3.6) | 4.4 (0.0, 10.0) | 6.4 (2.3, 10.5) | 1.5 (0.7, 2.3) | 5.0 (3.2, 6.7) |
|  | Weighted (95% CI)** | | 3.1 (1.9, 4.3) | 9.0 (0.0, 21.7) | 6.2 (0.8, 11.6) | 1.4 (0.3, 2.6)*** | 4.8 (2.2, 7.4) |
| 4-week (face-to-face respondents only) | Total cases | | 128 | 49 | 34 | 28 | 17 |
|  | Crude (95% CI) | | 4.0 (3.8, 4.2) | 6.6 (4.9, 8.2) | 4.1 (2.9, 5.3) | 3.5 (2.4, 4.5) | 2.1 (1.2, 2.9) |
|  | Weighted (95% CI)** | | 7.0 (4.0, 9.9) | 9.2 (1.3, 17.1) | 14.1 (4.9, 23.3) | 3.2 (1.9, 4.4)*** | 2.7 (0.7, 4.8) |
| Point | Total cases | | 27 | 11 | 4 | 4 | 8 |
|  | Crude (95% CI) | | 0.6 (0.4, 0.8) | 1.4 (0.6, 2.2) | 0.4 (0.0, 0.8) | 0.3 (0.0, 0.5) | 0.6 (0.2, 1.1) |
|  | Weighted (95% CI)** | | 0.8 (0.3, 1.3) | 1.5 (0.3, 2.6) | 1.5 (0.0, 3.6) | 0.2 (0.0, 0.4)*** | 0.6 (0.1, 1.0) |
| 2-week | Total cases | | 56 | 23 | 20 | 2 | 11 |
|  | Crude (95% CI) | | 1.3 (1.0, 1.6) | 2.9 (1.8, 4.1) | 2.2 (1.2, 3.1) | 0.1 (0.0, 0.3) | 0.9 (0.4, 1.4) |
|  | Weighted (95% CI)** | | 2.2 (2.0, 2.4) | 6.4 (0.0, 13.3) | 4.4 (1.3, 7.6) | 0.1 (0.0, 0.1)*** | 0.6 (0.2, 0.9) |
| 2-week diarrhoea prevalence among children below five years (Demographic and Health Survey) definition****** | Total cases | | 19 | 11 | 5 | 2 | 1 |
|  | Crude (95% CI) | | 3.7 (2.1, 5.4) | 9.0 (3.9, 14.1) | 20.8 (4.6, 37.1) | 0.6 (0.0, 1.5) | 1.8 (0.0, 5.4) |
|  | Weighted (95% CI)** | | 5.6 (1.9, 9.3) | 9.1 (1.8, 16.5) | 16.5 (1.4, 31.6) | 0.6 (0.0, 1.5)*** | 0.8 (0.0, 2.2) |

*CI (Confidence Interval)

**Weighted for age, gender, and urban/rural status

***Weighted for age and gender only

**** Nigerian population age proportion was used to standardize the incidence rates

*****Only urban

******the DHS definition**:** diarrhoea [1 or more loose stools] during the two weeks preceding the survey in children under five years of age [15–18]

**Table S4** Severity and symptoms among the 168 cases of acute gastrointestinal illness (AGI) in Ethiopia, Mozambique, Nigeria, and Tanzania, October 2020 – September 2021

| Severity | | | Number (%) | | | | |
| --- | --- | --- | --- | --- | --- | --- | --- |
|  |  |  | **Overall**  **(n=168)** | **Ethiopia**  **(n=51)** | **Mozambique**  **(n=41)** | **Nigeria**  **(n=37)** | **Tanzania**  **(n=39)** |
| Diarrhoea | Experienced any diarrhoea | | 156 (93.4) | 44 (86.3) | 41 (100) | 34 (91.9) | 37 (97.4) |
|  | Had blood in stool | | 19 (12.7) | 8 (18.2) | 5 (12.2) | 0 (0.0) | 6 (16.2) |
|  | Diarrhoea was constant/all-day long | | 10 (6.5) | 1 (2.4) | 1 (2.4) | 4 (11.8) | 4 (10.8) |
|  | Ave. maximum no. loose stools in 24 hours (95% CI) | | 4.0 (3.9, 4.2) | 4.4 (3.9, 4.9) | 3.7 (3.4, 4.0) | 4.3 (4.0, 4.5) | 3.8 (3.5, 4.1) |
| Vomiting | Experienced any vomiting | | 63 (37.5) | 28 (54.9) | 7 (17.1) | 9 (24.3) | 19 (48.7) |
|  | Vomiting was constant/all-day long | | 4 (7.0) | 0 (0.0) | 0 (0.0) | 3 (37.5) | 1 (5.5) |
|  | Ave. maximum no. times vomiting in 24 hours (95%CI) | | 2.9 (2.5, 3.3) | 3.2 (2.8, 3.7) | 2.3 (1.3, 3.3) | 2.6 (1.2, 4.0) | 2.8 (1.7, 3.8) |
| Mean (95% CI) duration of illness (days) | | Unweighted | 4.0 (3.5, 4.4) | 5.3 (4.4, 6.3) | 3.4 (2.4, 4.4) | 3.0 (2.3, 3.7) | 3.4 (2.6, 4.1) |
|  |  | Weighted* | 3.5 (3.1, 3.8) | 4.9 (4.1, 5.6) | 2.9 (2.2, 3.6) | 3.3 (2.5, 4.0)** | 3.9 (3.1, 4.7) |
| Cases with more than one episode of AGI in the previous 28 days | | | 87 (52.1) | 26 (51.0) | 25 (61.0) | 11 (30.5) | 25 (65.8) |
| Cases admitted to hospital overnight due to the illness | | | 10 (16.9) | 1 (6.3) | 3 (23.1) | 3 (33.3) | 3 (14.3) |
| Cases of AGI with at least one other person sick with AGI in their household | | | 41 (25.9) | 17 (33.3) | 13 (34.2) | 8 (21.6) | 3 (9.4) |
| Other associated symptoms | | | | | | | |
| Abdominal pain | | | 86 (51.2) | 21 (41.2) | 32 (78.0) | 9 (24.3) | 24 (61.5) |
| Stomach cramp | | | 57 (33.9) | 36 (70.6) | 8 (19.5) | 9 (24.3) | 4 (10.3) |
| Fever | | | 56 (33.3) | 23 (45.1) | 8 (19.5) | 14 (37.8) | 11 (28.2) |
| Headache | | | 34 (20.2) | 16 (31.4) | 13 (31.7) | 3 (8.1) | 2 (5.1) |
| Nausea | | | 20 (11.9) | 6 (11.8) | 7 (17.1) | 1 (2.7) | 6 (15.4) |
| Coughing | | | 15 (8.9) | 0 (0.0) | 5 (12.2) | 5 (13.5) | 5 (12.8) |
| Muscle/ body aches | | | 14 (8.3) | 2 (3.9) | 5 (12.2) | 5 (13.5) | 2 (5.1) |
| Chills | | | 9 (5.4) | 1 (2.0) | 1 (2.4) | 2 (5.4) | 5 (12.8) |
| Runny nose | | | 8 (4.8) | 0 (0.0) | 0 (0.0) | 2 (5.4) | 6 (15.4) |
| Sneezing | | | 6 (3.6) | 0 (0.0) | 3 (7.3) | 2 (5.4) | 1 (2.6) |
| Sore throat | | | 2 (1.2) | 0 (0.0) | 2 (4.9) | 0 (0.0) | 0 (0.0) |

*Weighted for age, gender, and urban/rural status

**Weighted for age and gender only

**Supplementary Table S5.** Odds of having acute gastrointestinal illness (AGI) by demographic characteristic in Ethiopia, Mozambique, Nigeria, and Tanzania, including handwashing (yes or no) and adjusted for all variables in the model*, for the time period February 2021 – September 2021 when handwashing information was collected (n=2611); significant values at α=0.05 are in bold

| Demographic Characteristic | Coefficient | Odds Ratio | 95% Confidence Interval |
| --- | --- | --- | --- |
| Gender | | | |
| Male | 0.21 | 1.23 | 0.78, 1.96 |
| Female | Ref. | Ref. | Ref. |
| Age (years) | | | |
| 0-4 | **1.24** | **3.46** | **1.89, 6.35** |
| 5-14 | 0.84 | 2.32 | 0.91, 5.91 |
| 15-59 | Ref. | Ref. | Ref. |
| 60+ | -0.27 | 0.77 | 0.29, 2.02 |
| Wealth index quintile | | | |
| Lowest | 0.41 | 1.51 | 0.80, 2.86 |
| Second & Middle | Ref. | Ref. | Ref. |
| Fourth | **1.07** | **2.91** | **1.64, 5.17** |
| Highest | 0.37 | 1.45 | 0.75, 2.86 |
| Residence | | | |
| Urban | Ref. | Ref. | Ref. |
| Rural | 0.45 | 1.57 | 0.96, 2.55 |
| Employment status of the main earner in the household | | | |
| Working | 0.16 | 1.17 | 0.69, 1.99 |
| Not working | Ref. | Ref. | Ref. |
| No. people sleeping per room | | | |
| ≤3 | Ref. | Ref. | Ref. |
| >3 | 0.30 | 1.35 | 0.76, 2.40 |
| Season | | | |
| Dry** | Ref. | Ref. | Ref. |
| Wet*** | 0.26 | 1.30 | 0.81, 2.09 |
| Country | | | |
| Nigeria | Ref. | Ref. | Ref. |
| Ethiopia | **1.50** | **4.49** | **1.98, 10.16** |
| Mozambique | **1.61** | **5.01** | **2.26, 11.09** |
| Tanzania | **0.83** | **2.29** | **1.08, 4.84** |
| Method of data collection | | | |
| Web-survey | **1.08** | **2.95** | **1.59, 5.47** |
| Face-to-face | Ref. | Ref. | Ref. |
| Hand washing with detergent and hand rubbing for 20 seconds | | | |
| Yes | 0.13 | 1.14 | 0.67, 1.93 |
| No | Ref. | Ref. | Ref. |

*This is the same as the model shown in Table 5, with the addition of the variable “Have you been washing your hands by applying any detergent/disinfectant solution with rubbing hands together for 20 seconds? (yes, no)”

**Dry season (Ethiopia: October 1 – May 31; Mozambique: April 1 – September 30; Nigeria: November 1 – March 31; Tanzania: June 1 – October 31)

***Wet season (Ethiopia: June 1 – September 30; Mozambique: October 1 – March 31; Nigeria: April 1- October 31; Tanzania: November 1 – May 31)

**Supplementary Table S6.** Odds of having acute gastrointestinal illness (AGI) by demographic characteristic in Ethiopia, Mozambique, Nigeria, and Tanzania, including handwashing (yes, no, unknown) and adjusted for all variables in the model*, October 2020 – September 2021 (n=4533); significant values at α=0.05 are in bold

| Demographic Characteristic | Coefficient | Odds Ratio | 95% Confidence Interval |
| --- | --- | --- | --- |
| Gender | | | |
| Male | 0.08 | 1.08 | 0.76, 1.54 |
| Female | Ref. | Ref. | Ref. |
| Age (years) | | | |
| 0-4 | **1.52** | **4.58** | **2.95, 7.10** |
| 5-14 | 0.73 | 2.08 | 0.99, 4.37 |
| 15-59 | Ref. | Ref. | Ref. |
| 60+ | 0.06 | 1.06 | 0.56, 2.01 |
| Wealth index quintile | | | |
| Lowest | 0.37 | 1.44 | 0.92, 2.27 |
| Second & Middle | Ref. | Ref. | Ref. |
| Fourth | **0.45** | **1.57** | **1.01, 2.42** |
| Highest | 0.04 | 1.04 | 0.63, 1.70 |
| Residence | | | |
| Urban | Ref. | Ref. | Ref. |
| Rural | **0.41** | **1.51** | **1.04, 2.19** |
| Employment status of the main earner in the household | | | |
| Working | 0.19 | 1.21 | 0.79, 1.85 |
| Not working | Ref. | Ref. | Ref. |
| No. people sleeping per room | | | |
| ≤3 | Ref. | Ref. | Ref. |
| >3 | 0.27 | 1.31 | 0.85, 2.03 |
| Season | | | |
| Dry** | Ref. | Ref. | Ref. |
| Wet*** | 0.23 | 1.25 | 0.87, 1.81 |
| Country | | | |
| Nigeria | Ref. | Ref. | Ref. |
| Ethiopia | **1.58** | **4.87** | **2.72, 8.72** |
| Mozambique | **1.56** | **4.76** | **2.67, 8.48** |
| Tanzania | **0.97** | **2.64** | **1.49, 4.67** |
| Method of data collection | | | |
| Web-survey | **0.83** | **2.28** | **1.38, 3.79** |
| Face-to-face | Ref. | Ref. | Ref. |
| Hand washing with detergent and hand rubbing for 20 seconds | | | |
| Yes | -0.03 | 0.97 | 0.66, 1.43 |
| No | -0.24 | 0.79 | 0.46, 1.33 |
| Unknown | Ref. | Ref. | Ref. |

*This is the same as the model shown in Table 5, with the addition of the variable “Have you been washing your hands by applying any detergent/disinfectant solution with rubbing hands together for 20 seconds? (yes, no, unknown)” and treating the prior period (October 2020 to January 2021) where the variable was not collected as unknown

**Dry season (Ethiopia: October 1 – May 31; Mozambique: April 1 – September 30; Nigeria: November 1 – March 31; Tanzania: June 1 – October 31)

***Wet season (Ethiopia: June 1 – September 30; Mozambique: October 1 – March 31; Nigeria: April 1- October 31; Tanzania: November 1 – May 31)

**Supplementary Table S7.** Odds of having acute gastrointestinal illness (AGI) by demographic characteristic in Ethiopia, Mozambique, Nigeria, and Tanzania, including handwashing frequency and adjusted for all variables in the model*, for the time period February 2021 – September 2021 when handwashing information was collected (n=2611); significant values at α=0.05 are in bold

| Demographic Characteristic | Coefficient | Odds Ratio | 95% Confidence Interval |
| --- | --- | --- | --- |
| Gender | | | |
| Male | 0.58 | 1.79 | 0.96, 3.34 |
| Female | Ref. | Ref. | Ref. |
| Age (years) | | | |
| 0-4 | **1.00** | **2.72** | **1.09, 6.79** |
| 5-14 | 0.80 | 2.23 | 0.59, 8.40 |
| 15-59 | Ref. | Ref. | Ref. |
| 60+ | -0.72 | 0.49 | 0.14, 1.73 |
| Wealth index quintile | | | |
| Lowest | **1.** | **3.08** | **1.19, 8.01** |
| Second & Middle | Ref. | Ref. | Ref. |
| Fourth | **1.52** | **4.57** | **2.03, 10.26** |
| Highest | 0.69 | 2.00 | 0.83, 4.83 |
| Residence | | | |
| Urban | Ref. | Ref. | Ref. |
| Rural | **0.76** | **2.13** | **1.14, 3.97** |
| Employment status of the main earner in the household | | | |
| Working | -0.02 | 0.97 | 0.50, 1.89 |
| Not working | Ref. | Ref. | Ref. |
| No. people sleeping per room | | | |
| ≤3 | Ref. | Ref. | Ref. |
| >3 | 0.18 | 1.20 | 0.48, 2.99 |
| Season | | | |
| Dry** | Ref. | Ref. | Ref. |
| Wet*** | 0.27 | 1.31 | 0.69, 2.50 |
| Country | | | |
| Nigeria | Ref. | Ref. | Ref. |
| Ethiopia | **1.86** | **6.43** | **1.44, 28.07** |
| Mozambique | **1.89** | **6.43** | **1.44, 28.70** |
| Tanzania | **1.45** | **4.25** | **1.09, 16.62** |
| Method of data collection | | | |
| Web-survey | **0.87** | **2.39** | **1.08, 5.30** |
| Face-to-face | Ref. | Ref. | Ref. |
| Hand washing frequency: average per day in the previous 2-week period | -0.02 | 0.98 | 0.95, 1.01 |

*This is the same as the model shown in Table 5, with the addition of the variable “How many times (on average) do you wash/sanitize your hands per day within the last two weeks? (number)”

**Dry season (Ethiopia: October 1 – May 31; Mozambique: April 1 – September 30; Nigeria: November 1 – March 31; Tanzania: June 1 – October 31)

***Wet season (Ethiopia: June 1 – September 30; Mozambique: October 1 – March 31; Nigeria: April 1- October 31; Tanzania: November 1 – May 3
